# “Intra-operative assessment of leg length discrepancy with anterior approach total hip replacement: a comparison between standard table, position table with and without intra-operative radiographs”

**DOI:** 10.1007/s00264-025-06411-9

**Published:** 2025-02-14

**Authors:** Edoardo Viglietta, Simone Fenucci, Raffaele Iorio, Ariane Parisien, George Grammatopoulos, Paul Roy Kim, Paul Edgar Beaulè

**Affiliations:** 1 Orthopaedic Unit, S. Andrea Hospital, University of Rome “La Sapienza”, Rome, Italy; 2 Orthopedic Surgeon University of Ottawa/The Ottawa Hospital, Ottawa, Ontario Canada

**Keywords:** THA, DAA, LLD, Leg length, Fluoroscopy, Orthopaedic table, Intra-operative

## Abstract

**Purpose:**

Post-operative LLD is a major concern after THA. The anterior approach on a standard table allows surgeons for a direct control of the leg length. Intra-operative radiography (IR) helps in assessment of hip biomechanics and anatomic parameters. The aim of this study is to evaluate the LLD after THA through anterior approach with or without a position table and with or without the use of intra-operative radiographs. The hypothesis is that leg length may be better control when IR and a standard table are used.

**Methods:**

This is a single-centre retrospective comparative cohort study of three matched groups of 80 patients receiving anterior approach THA with three different techniques (Group A: positioning table with IR; Group B: standard table with IR; Group C: standard table without IR). Pre-operative and post-operative LLD was calculated. Age, sex, BMI, acetabular cup and femoral stem size, operative time, and blood loss were recorded.

**Results:**

In Group A, 15 patients (19%) had a LLD greater than 5 mm, and two patients (2,5%) had a LLD greater than 10 mm. In Group B, 20 patients (25%) had a LLD greater than 5 mm, and two patients (2,5%) had a LLD greater than 10 mm. In Group C, 16 patients (20%) had a LLD greater than 5 mm, and three patients (3,7%) had a LLD greater than 10 mm. No statistically significant differences were found for LLD > 5 mm, for LLD > 10 mm, nor for the mean LLD between the three groups (*p  > 0.05*). Mean operative time was statistically longer in Group B (*p  < 0.05*).

**Conclusion:**

Neither the use of a standard/positioning table neither the use of IR seemed to be superior in restoring leg length after anterior approach THA. Together with the contradictory results in literature, findings of the current study indicate that no technique is clearly superior to one other and surgeons’ experience may play the most relevant role.

## Introduction

Postoperative leg length discrepancy (LLD) is a major concern after total hip arthroplasty (THA) [1]. Even though a true definition of LLD is not worldwide accepted, LLD is known to cause poor patient satisfaction, with gait impairment, back pain, component instability, and degenerative changes on contralateral hip and ipsilateral knee, especially if greater than 1 mm [1].

Over the past years, the anterior approach (AA) has gained increased popularity due to a lower risk of dislocation, an expected early functional recovery, a lower soft tissue damage, and an easier control of leg length and hip biomechanics parameters [2–6]. According to the AOA National Joint Register AA represents the second most common approach with the 30% of patients receiving primary THA for osteoarthritis through a DAA [7].

The AAcan be performed with a positioning or a standard operating roomtable. The positioning table eases the surgical procedures reducing the number of required assistants, reducing the soft tissue release for the femoral exposure, and reducing the operative time [2, 8–10]. However, the positioning table does not allow surgeons for direct leg length assessment, and requires additional costs [11–13].

Intra-operative radiography (IR) may help surgeons to assess and restore the proper biomechanics of the hip during AA by the evaluation of leg length, femoral and acetabular offset, and component position. However, asymmetry of the beam, femoral abduction or rotation of the pelvis may affect the reliability of IR, and the actual benefit to prevent postoperative LLD has not yet been clearly defined [14–16].

The aim of this study is to evaluate the LLD after THA through AA with or without a position table and with or without the use of IR. The hypothesis is that leg length may be better control when IR and a standard table are used.

## Methods

This is a single-centre retrospective comparative cohort study. Patients undergone to unilateral or bilateral primary THA through an AA at our institution since January 2023 to December 2024 were considered eligible for the study.

Three different experienced surgeons performed AA THA at Our Institute with three different techniques: (1) AA using positioning table with IR, (2) DAA using standard table with IR, and (3) AA using a standard table without IR.

The consecutive series of patients of each surgeon was considered eligible for the study and identified from the Institutional electronic database. Patients were matched according to age, sex and BMI and subsequently three matched group of 80 patients were obtained for each technique (Group A: AA using positioning table with IR; Group B: AA using standard table with IR; Group C: AA using a standard table without IR).

Exclusion criteria included: (1) pre-existing abnormalities severely affecting anatomy of the native hip; (2) pre-existing LLD greater than 1 cm; (3) contralateral severe osteoarthritis or previous surgery impairing proper leg length; (4) revision hip arthroplasty; (5) any intra-operative complication responsible for LLD; and 5) lack of radiological follow-up.

All patients received the same Hueter interval for anterior approach. Patients were positioned supine on the positioning or standard operating room table. Skin incision was performed starting 2 cm below and lateral from the anterior iliac spine and extending distally for 8–10 cm towards the fibula head. The interval between the tensor fasciae latae (TFL) and sartorius was developed in a blunt fashion, protecting the lateral femoral cutaneous nerve protected. The ascending branches of the lateral circumflex artery were coagulated. The gluteus medius was retracted laterally and the rectus femoris was elevated from the hip capsule and then retracted medially placing two Hohmann retractors superiorly and inferiorly to the femoral neck. The anterior capsule was excised to expose the joint in the Group A and C, while in the Group B anterior capsule was incised, elevated and then reinserted at the end of the procedure according to the surgeon’s preference. The osteotomy of the femoral neck and the removal of the femoral head were performed taking care to protect the surrounding soft tissues. Release of the ilio-pubic ligament was performed anteriorly and release of the digitae fossae was performed postero-laterally to obtain proper femoral exposure. Acetabular reaming was performed in the standard fashion and trial and final acetabular cup were positioned. Femoral preparation was performed in the Group A by femoral adduction, extension and maximal external rotation on the positioning table, while in Group B and C on a standard operating table with the lower limb in a figure-four. position under the contralateral limb. With the trial femoral component in site, IR for an antero-posterior view of the pelvis was performed in the Group A and B. LLD was checked and final components were positioned accordingly. In group C, LLD was assessed intra-operatively with the surgeon palpating and comparing both malleoli.

Before surgery a preoperative templating was performed for all the patients using the OrthoView software (OrthoView Materialise HQ, Belgium). Femoral osteotomy level, implant size and positioning, and leg length correction were assessed. All patients were discharged the same day or the day after surgery. The same post-operative protocol with full weight-bearing and no mobility restriction was adopted. A post-operative antero-posterior X-Ray of the pelvis was performed at the four weeks follow-up visit.

**Fig. 1:**
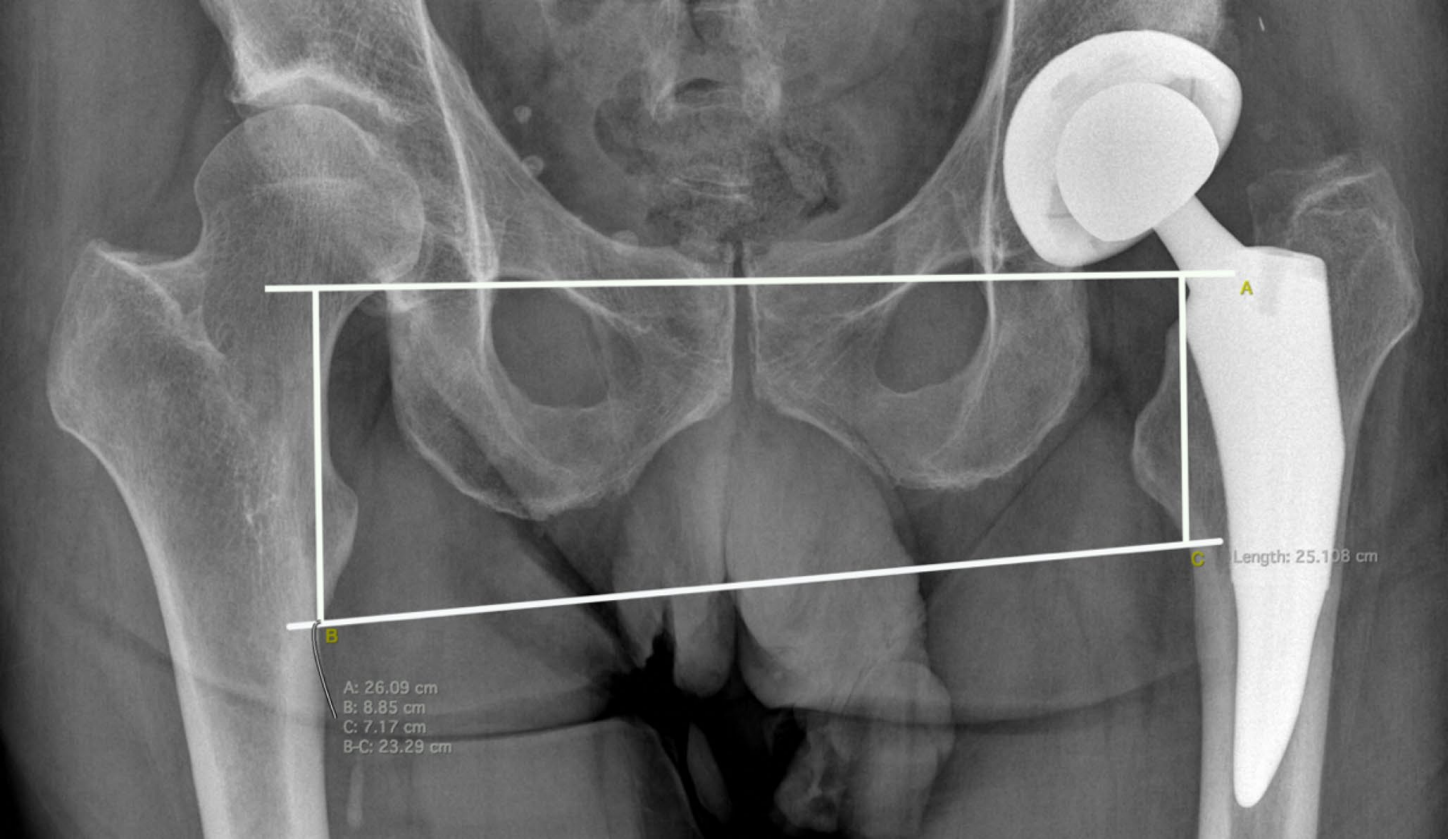
LLD measurement according to Ranawat

Pre-operative and post-operative LLD was calculated by a third surgeon, not involved in the surgical procedures and blinded to the three study groups. The interteardrop reference line method described by Ranawat et al. [17] was adopted (Fig. 1). A reference line connecting the inferior margin of the radiographic teardrop was drawn. The distance between the reference line and a perpendicular line from the edge of the lesser trochanter was measured on each side. The difference in length between the surgical and nonsurgical legs was recorded as the LLD.

The following data were collected for each patient: age, sex, BMI, LLD, acetabular cup and femoral stem size, operative time, and blood loss.

The achieved power of the study according to the post hoc power analysis with a total sample size of 240 hips, medium effect size, and alpha = 0.05 was 96%. Descriptive statistics are presented as mean ± standard deviation (SD). All parameters were tested for normality to compare normal variables; ANOVA one-way test was used. Otherwise, the Wilcoxon signed-rank tests were used. Fisher’s exact test was used to look for any association between LLD and other variables.

## Results

The study included 240 patients (113 females and 127 males). The mean age was 67 years in Group A, B, and C. The mean BMI was 28, in Group A, B, and C. Demographic data are reported in Table 1.

**Table 1:** Demographic matached population

	**Group A**	**Group B**	**Group C**
**Sex F: M (% Male)**	38:42 (53%)	39:41 (51%)	36:44 (55%)
**Mean BMI**	27,6	27,6	27,6
Min	20	20	20
Max	43	43	43
**Mean Age**	66,9	66,9	66,8
Min	48	50	49
Max	87	87	86
**Height**	172,1	169,5	170,5
Min	152	152	155
Max	187	192	191
**Weight**	81,8	79,4	80,1
Min	50	50	52
Max	118	111	117

In Group A, 15 patients (19%) had a LLD greater than 5 mm (4 shortening and 11 lengthening), two patients (2,5%) had a LLD greater than 10 mm, and zero patients had a LLD greater than 15 mm. The mean LLD was 3,6 mm.

In Group B, 20 patients (25%) had a LLD greater than 5 mm (13 shortening and 7 lengthening), two patients (2,5%) had a LLD greater than 10 mm, zero patients had a LLD greater than 15 mm. The mean LLD was 4,3 mm.

In Group C, 16 patients (20%) had a LLD greater than 5 mm (7 shortening and 9 lengthening), three patients (3,7%) had a LLD greater than 10 mm, zero patients had a LLD greater than 15 mm. The mean LLD was 3,8 mm.

**Fig. 2:**
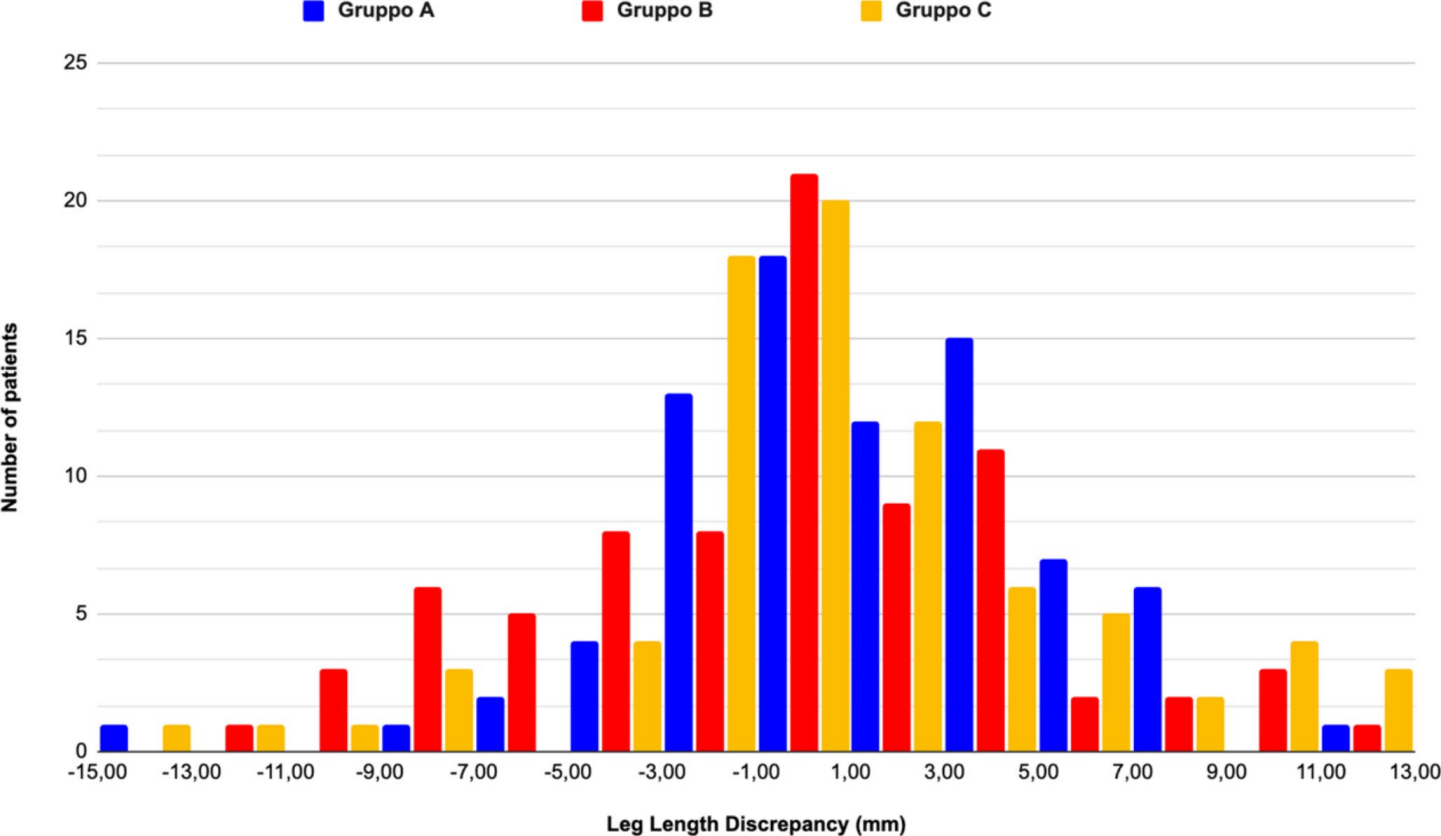
LLD distribution in the three different Groups

No statistically significant differences were found for LLD > 5 mm, for LLD > 10 mm, nor for the mean LLD between the three groups (*p #x2009;> 0.05*) (Table 2; Fig. 2).

**Table 2:** Overall comparison between groups

	**Group A**	**Group B**	**Group C**
**Mean LLD mm** **LLD > 5 n(%)**	3.6 15 (19)	4.3 20 (25)	3.8 16 (20)
**LLD > 10 n(%)**	2 (2.5)	2 (2.5)	3 (3.7)
**Lengthening**	11 (14)	7 (9)	9 (11)
**Shortening**	4 (5)	13 (16)	7 (9)

Mean operative time was statistically longer in Group B (mean operative time was 54, 80, and 59 min in Group A, B, and C respectively: *p  <  0.05*).

No statistically significant correlation was found between LLD and age, sex, BMI, operative time or the use of a specific table or IF (*p  >  0.05*).

## Discussion

The main finding of the current study is that the use of a positioning table and/or IR do not significantly impact on the accuracy of restoring proper leg length in patients undergoing THA through a AA. No statistically significant correlation between LLD and the use of a positioning table and/or IR or any demographic parameter has been found. Thus, the hypothesis of the study has been rejected. Operative time is statistically longer when both a standard operating table and IR are used.

LLD after THA has been associated to poor patients’ satisfaction and increased litigation toward operating surgeons [4, 18]. The true threshold between acceptable and unacceptable levels of LLD is unclear [19], but shortening greater than 10 mm and lengthening greater than 6 mm have been reported to be perceived by patients and the percentage of patients who note their LLD is up to 32% [20]. Many intra-operative techniques are commonly adopted to mitigate the risk of LLD, but it has been reported that a mean LLD of 3 to 17 mm still occurs after unilateral THA [1].

In this regard, AA may provide the benefit of an easier assessment of LLD intra-operatively by direct palpation of medial malleoli or by the use of IR. When a standard operating table is used for AA, surgeons are allowed to check for LLD (by palpation of the medial malleoli and iliac spines), and for implant stability [21]. When a positioning table is used and the pelvis is stabilized by the perineal support, standardized IR enables the assessment of LLD [22]. Furthermore, a positioning table may simplify the procedure, especially during the DAA learning curve for better exposure of femur [23].

Comparing AA with and without a positioning table on 266 primary THAs, Moslemi et al. [24] reported not statistical differences in LLD, implant positioning and complications rate between groups. However, several surgeons participated to the study and more junior surgeons were involved in the standard table group. Similarly, more cementless stem were used in the standard table group. All of these represent possible bias and should be considered in assessing their findings. Conversely, Wernly et al. [25] performed a matched-control study comparing 75 patients treated through AA with positioning table and 75 patients treated through AA with a standard table by the same senior surgeon. They reported a two-fold incidence of 5 mm LLD with a positioning Table (10% vs. 20%), and a 2.5% rate of 10 mm LLD with a positioning table while no cases of 10 mm LLD without a taction a table. They concluded that the standard table group present a significantly more accurate restoration of leg length and those findings may find confirmation in recent literature. Batailler et al. [26] reported a mean LLD of 2 mm without a positioning table, while a higher LLD (up to 7 mm) has been found with a positioning table by several Authors [27–29]. According to the results of the current study the mean LLD was similar to that reported in recent literature, but no statistically significant differences are present between groups for 5 mm, 10 mm and 15 mm LLD.

Recent studies have largely advocated for the use of intra-operative fluoroscopy(IF) in AA [18, 30]. A significative reduction in post-operative LLD has been reported by Leucht et al. comparing AA with IF and posterior approach without IF (i.e. (0.7 vs. 2.7 mm) [18]. Conversely, contradictory results have been reported by Bingham et al. who did not find significant difference in postoperative LLD comparing AA with and without IF [31]. However, in their study inter-surgeon variables were not controlled. Two different surgeons performed THA of both the groups, and this certainly represents a bias of their investigation.

Furthermore, IF or OR presents some limitations which should be considered. Firstly, LLD is assessed in relation of specific anatomic landmarks (e.g. the inferior ischial line, the inferior interteardrop line, the superior aspect of the lesser trochanter or tip of the greater trochanter) and this measurement may be impaired by radiograph asymmetry or adduction and rotation differences in femoral and pelvis positions. Secondly, IF and IR may be responsible for a longer operative time as confirmed by the current study. Lastly, IF and IR certainly cause radiation exposure for surgeons and patients with subsequent radiological risks. For these reasons, robust and true evidences of the advantages of IF are required to suggest the widely adoption of IF in AA THA.

Blum et al. [32] assessed the use of IF with DAA and posterior approach for THA. A statistically significant lower rate of LLD in patients treated through a AA with IF when compared to patients treated through a AA without IF or through a posterior approach without IF. They concluded that the use of IF may be beneficial in reducing the risk of LLD > 5 mm, and > 10 mm. With the widespread use innovative technologies, O’Leary et al. [33] compared the use of standard IF with digital IF in DAA. According to their findings, digital IF showed to be significantly more accurate in respecting the leg lengthening of the pre-operative planning when compared to standard IF. Nevertheless, a satisfying restoration of leg length has been reported also with traditional and less expensive methods by several Authors [34, 35]. According to those findings, IF represents a reliable tool surgeons may use during AA THA to restore patients’ leg length. Conversely, results of the current study did not show a significant reduction in LLD when IF is used, likely indicating that surgeons’ experience and preference still have the main role in guaranteeing a satisfying result after AA THA.

The main strength of the study consists in the simultaneously evaluation of the positioning table and IF in avoiding LLD on a matched population of patients undergoing THA through AA. All the operations were performed by senior surgeon with decades experience in AA and this represents the second strength of the study.

This study also presents some limitations. Firstly, patients were not clinically evaluated and is not possible to correlate the radiological finding of LLD to any symptom perceived by patients. However, this was not the intent of the current study which aimed to assess the accuracy of different strategies to prevent LLD in AA. Secondly, three different senior surgeons employing three different techniques performed surgical procedures in the current study. Even though each surgeons employed a specific technique – responsible for patients’ allocation in three different groups - the Authors acknowledge that extrapolation of these results to all surgeons is speculative.

## Conclusion

Neither the use of a standard/positioning table neither the use of IR seemed to be superior in restoring leg length after AA THA. Together with the contradictory results in literature, findings of the current study indicate that no technique is clearly superior to one other and surgeons’ experience may play the most relevant role. Further research evaluating LLD during the surgeon learning curve could be useful to better elucidate which technique allows for a better control of leg length in AA THA.

## Data Availability

No datasets were generated or analysed during the current study.
